# Trained innate immunity, COVID-19 therapeutic dilemma, and fake science

**DOI:** 10.6061/clinics/2020/e2124

**Published:** 2020-07-06

**Authors:** José Ernesto Belizário

**Affiliations:** Hospital das Clinicas HCFMUSP, Faculdade de Medicina, Universidade de Sao Paulo, Sao Paulo, SP, BR

## TRAINED INNATE IMMUNITY AND COVID-19

When we get sick, our body’s first line of defense, the immune cells, responds and stores a memory of the pathogen; this is called immunological memory (1). Two types of functionally distinct memories have been described, *i.e.*, innate memory (non-specific) and acquired memory (specific). There are two ways by which T and B cells form an acquired immunological memory. The first is based on genetic mechanisms that involve the recombination of membrane receptor genes (TCR) and selection of T cell clones capable of perfectly recognizing self and non-self antigens (*e.g.* proteins belonging to pathogens). The second, is based on the recombination of immunoglobulin genes (antibody genes) that recognize the proteins expressed by pathogens and the selection of memory B cell clones. Memory B cells express membrane receptors (BCR)—or bound antibody molecules with high affinity—that recognize the pathogens in the event of a second infection. However, the big question is, “how are antibodies produced against an infectious pathogen or vaccine?” First, dendritic cells phagocytize and degrade the pathogen and present its pieces in the form of epitopes (protein fragments) to CD4+ T lymphocytes. Then, helper CD4+ T cells communicate with lymphocytes B, which initiate the production of different classes of antibodies (humoral soluble response) against these epitopes. B lymphocyte clones die at the end of the infection and only few clones that contain the code (memory) for the synthesis of specific antibodies remain. Another population of lymphocytes, called effector CD8+ T lymphocytes (cellular response), also recognizes pathogen-related antigens in infected cells. These cytotoxic lymphocytes attack the pathogens by releasing cytokines, toxins, and enzymes that lead to cell death via apoptosis and necroptosis. These cytotoxic lymphocytes also die at the end, and only a few clones survive. The survivors are programmed to become memory CD8+ T lymphocytes that would recognize the pathogen in the event of a second infection.

How is memory in CD8+ T cells formed chemically? What we know is that cytosine and guanine (CpG)-rich regions in the promoters of genes encoding for various proteins, such as transcription factors, cytotoxic proteins, and cytokines involved in lymphocyte activation, undergo chemical modifications (methylation and demethylation, *i.e.*, addition or removal of methyl groups). Such modifications form a silencing or activating on/off switch for the transcription of immune response genes. Histone proteins that bind DNA molecules also undergo methylation and acetylation at their lysine and arginine residues. These types of chemical changes are referred to epigenetic and non-genetic (non-hereditary) modifications, as they do not cause any changes (mutations) in the DNA molecule, nor are they transmitted to the next generation. Therefore, children need to receive vaccines that protected their parents from pathogen, for example, the measles vaccine, to develop their own immune responses.

Bone marrow progenitor myeloid cells that give rise to blood leukocytes, such as neutrophils, monocytes, and natural killer cells (NKs), are the innate cells participating in the non-specific innate response and trained immunity (2). Studies have shown that monocytes and macrophages are “educated or trained” during the first infection, and thus they acquire the ability to fight more effectively in subsequent infections. Monocytes are trained through stimulation with lipopolysaccharide (LPS)—a gram-negative bacterial membrane protein—or beta-glucan, a component of the fungal cell wall. For example, the bacillus Calmette-Guérin (BCG) vaccine can increase the production of pro-inflammatory cytokines, such as tumor necrosis factor (TNF), interleukin-1 (IL-1), and IL-6, up to 5 times on second contact with the pathogen. This type of immunological memory or epigenetic programming to a pre-activated state allows the generation of a sustained and more effective non-specific response, even after years, although in the protocols of these studies, the innate immunity was evaluated after 3 months (2).

Biochemical analyses on chromatin showed that trained monocytes are characterized by an increase in histone 3 acetylation, in particular H3K27Ac and H3K4me3 as well as by an increase in the metabolism of glucose (glycolysis) and glutamine (glutaminolysis), and by high levels of fumarate, a metabolite of the tricarboxylic acid cycle or Krebs cycle (2). It has also been observed that after the training of human monocytes with *Candida albicans* (a human opportunistic pathogen) beta-glucan, the induced innate immunity protects not only against fungi, but also against bacteria, viruses, and parasites (2). In addition, the training of human monocytes by *Saccharomyces cerevisiae* (another human opportunistic pathogen) chitin greatly increased their ability to eliminate microbes such as *Candida albicans*, *Staphylococcus aureu*s (a gram-positive bacterium), and *Escherichia coli* (a gram-negative bacterium) in comparison with untrained human monocytes. More interesting, the non-specific effects of BCG vaccination improved the effects of low-efficiency vaccines, such as the vaccine against typhus—caused by *Salmonella typhi* (http://www.clinicaltrials.gov, number NCT02175420)—or the influenza vaccine. This protective effect has been known for decades in many countries (*e.g.* in Denmark and South Africa) where the BCG vaccine is administered to babies a few days after birth. In these countries, there was a 38-70% reduction in infant mortality associated with pneumonia and sepsis (3). However, to the best of our knowledge, none of the studies have determined if a similar phenomenon occurs in vaccinated babies in Brazil.

Various clinical trials are underway to evaluate trained immunity through BCG vaccination in healthy volunteers under the coordination of Dr. Mihail Netea (Radboud University Medical Center, Nijmegen, the Netherlands). Clinical trials BRACE (http://www.clinicaltrialgov, number NCT04327206) and BCG-corona (http://www.clinicaltrial.gov, number NCT04328441) are employing large cohorts of health professionals in the Netherlands, Denmark, Germany, England, France, Tanzania, Uganda, Colombia, and Uruguay (3). The objective is to demonstrate whether immunization with BCG vaccines produced using different strains and titers of the bacillus Calmette-Guérin—the vaccine against tuberculosis—can protect these professionals against SARS-CoV-2 infection (3). A similar study will be carried out in Brazil (http://www.clinicaltrial.gov, number NCT04369794). Dr. Netea said that preliminary results showed that in a large number of human volunteer cohorts, the BCG vaccine induced trained immunity (>50%; as stated in https://www.youtube.com/watch?v=3W36p40poLs). It is expected that the volunteers, if infected, will respond mildly or asymptomatically to the SARS-CoV-2 infection. The scientific basis for this hypothesis comes from studies undertaken on human volunteers previously immunized with the live BCG vaccine and then with the vaccine for the yellow fever virus that causes a hemorrhagic disease (4). It has also been verified through epidemiological and observational clinical studies that the number of deaths caused by coronaviruses in low-income countries, such as India and some countries in Africa and the Americas, are significantly lower than those in countries with medium and high levels of economic development, such as Italy, Belgium, Holland, and the United States of America (5). Despite the presence of evidences regarding the efficacy of BCG, the latter countries have not adopted the universal policy of mandatory immunization against tuberculosis (6). Live or attenuated vaccines against measles and smallpox as well as the oral polio vaccine are also effective in inducing innate cross-protection against other unrelated viral infections. The hypothesis that all vaccinated children are protected or are less likely to develop severe symptoms of the SARS-CoV-2 has been contested by many investigators (7,8). Therefore, we need to wait for the results of the clinical trials that are currently underway.

In Brazil, epidemiological data on tuberculosis, published by the Ministry of Health on March 2019, indicates that the incidence of the disease (30-35 cases/100 thousand inhabitants) has not changed in the last 10 years (9). Rio de Janeiro, Amazonas, Pará, Roraima, and Acre are the states with a tuberculosis incidence higher than the national average. In addition, mortality is higher than the national average (2.2 deaths /100 thousand inhabitants) in Rio de Janeiro, Amazonas, Pernambuco, Rio Grande do Sul, Pará, Maranhão, Rio Grande do Norte, Ceará, and Acre. Vaccination, although recommended by the WHO (World Health organization) for vulnerable populations, is not routinely employed, and only newborns receive the vaccine in Brazil. since 2010, the Butantan Institute has stopped the production of oral BCG (live strain); now, it only produces the BCG vaccine formulated using the recombinant tuberculin protein. To our knowledge, there are no published articles or clinical evidences that show that BCG immunization protects against SARS-CoV-2 in Brazil. Is innate memory more effective against SARS-CoV-2 than acquired memory? Could BCG vaccination be a more promising therapeutic alternative than chloroquine? These are the questions that need to be addressed.

## WHY DO RESEARCH TRIALS FAIL?

Scientific knowledge is accepted or rejected based on measures of probabilities. How evidence is transformed into scientific knowledge depends on statistical methods that define whether certain types of interferences (errors, biases, or confounding factors)—that occur either randomly or systematically—are leading to relevant clinical outcomes in patient cohorts (10). Chloroquine has been used in the prevention and treatment of malaria since 1947 (11). Its clinical use in the treatment of rheumatoid arthritis and lupus erythematosus has been approved using pre-established protocols and doses based on the disease stage and the clinical conditions of the patient. The side effects of chloroquine, such as retinopathy and ventricular arrhythmia, are well-known, and are rarely reported by patients (11). Chloroquine should not be used in the absence of any medical supervision in patients with diabetes and heart problems, neither in people over 65 who—among other problems—may have reduced kidney function. Therefore, any clinical study aimed at assessing the therapeutic effects of chloroquine should not include volunteers or patients having such comorbidities; therefore, these caveats should be included as a part of the trial protocol when establishing the inclusion and exclusion criteria. A previous study in a small patient cohort showed evidence that chloroquine could exhibit therapeutic effects in patients with COVID-19 (12). The journal *The Lancet*, in May 2020, published the results of an observational, longitudinal, and retrospective clinical study based on medical records of COVID-19 patient cohorts treated across 6 countries and 671 hospitals, with different technical capabilities and diverse drug protocols (13). The results suggested that chloroquine and hydroxychloroquine—and their combination with azithromycin—did not result in any clinical benefit; on the contrary, they worsened the condition of the patients. However, in these studies, patients with several comorbidities were evaluated, including those with cardiovascular disease (including congestive heart failure and history of heart failure arrhythmia), current or previous smoking history, history of hypertension, diabetes, or hyperlipidemia, or chronic obstructive pulmonary disease (COPD). These trials also reported that 47% of the treated patients needed admission to an intensive care unit (ICU) and assisted ventilation (severe case of the disease) against only 5% in the control group. The authors concluded that all underlying diseases (comorbidities)—considered as confounding factors—influenced the mortality rate; this was the most relevant outcome of the study. The secondary outcome of interest was related to ventricular arrhythmia. It was mentioned that, among the 81 000 patients in the control group, a small population (16%, 14 300) had a history of heart disease, whereas 890 (1%) suffered from *de novo* ventricular arrhythmia, along with in-hospital treatment, and survived. Of the 14 800 patients in the treatment group, 10 600 died. A large number of patients in the non-survival population (33%, 3 500 patients) had a history of cardiovascular disease. These were the patients for whom chloroquine treatment was not recommended because of the high risk of suffering from adverse effects, such as the prolonged QT interval and arrhythmia. In this group, as expected, 400 patients (3.7%) suffered from *de novo* ventricular arrhythmia (13). Whether this effect was observed before or during the treatment, was not specified by the authors. The episodes of *de novo* ventricular arrhythmia could be induced by several factors and clinical conditions; these include treatment with chloroquine, hydroxychloroquine, or a combination of chloroquine and azithromycin or that of hydroxychloroquine and azithromycin, and—finally—the pathology caused by SARS-CoV-2 (as it was observed in patients in the control group). I think that the study was not useful—or was partially useful—for assessing the cause and effect relationship of the medications because of the heterogeneity of the confounding factors. In fact, this paper was retracted a few days after its publication. Therefore, further studies are required to assess the effect of the tested medicaments in patients only having COVID-19 illness at the early phase, in which the drugs appear to exhibit the expected therapeutic benefits.

The uncertainties and dilemmas regarding COVID-19 and its treatment can be attributed to the fact that everything we know about this disease is still insufficient. There is no other way to prove the veracity of scientific findings without the replication of facts and experiences. Clinical trials in humans must be guided by the practice standards, norms, and rules established in the International Conference on Harmonization / Good Clinical Practices (ICH/GCP), while following the ethical principles of the Helsinki Declaration, proclaimed in June 1964 (http://www.wma.net), and the Hippocratic Oath, the origin of the modern medical ethics. To develop evidence-based effective public health strategies, all clinical protocols must be based on evidence, which is defined as the link between excellent scientific research and good clinical practices. Transparency in clinical trials begins with trial registration at the WHO International Clinical Trials Registry Platform (ICTRP), ClinicalTrials.gov; as detailed before, the Brazilian Clinical Trials Registry (ReBEC) is involved in this process in Brazil (14). Although scientists are widely trusted and feted for their discoveries, they are repeatedly required to reexamine their findings using new technological strategies and new knowledge. The randomized clinical study (RCT) is a scientific innovation; a way to draw better conclusions about cause and effect of medications or clinical procedures in matched and paired cohort groups. A double-blind randomized clinical study is another scientific innovation; a way to avoid the interference of patients and investigators on the results. In this context, the effects of the placebo (inert drug) must be tested, and the effectiveness of the drug/vaccine must be higher in the treatment group than that in the placebo group. Surrogate markers and secondary endpoints are commonly used in clinical trials to anticipate absolute primary outcomes, which will result in beneficial or adverse effects in the patients. Equally important are the adoption of new strategies to analyze the data and draw scientific conclusions. Robust statistical methods and well-designed experiments are the fundamental requirements for testing novel treatments and repurposing existing drugs. The studies in progress—to evaluate the therapeutic effects of BCG and chloroquine in COVID-19—need to continue to answer these important questions and to reinstate peoplés trust in science. In the meantime, many lives may be lost. Therefore, we must exercise caution as to what types of evidence we can accept and share, and the types of arguments and reasons to publish any fact on a social network.
